# Inequalities in life expectancy in six large Latin American cities from the SALURBAL study: an ecological analysis

**DOI:** 10.1016/S2542-5196(19)30235-9

**Published:** 2019-12

**Authors:** Usama Bilal, Marcio Alazraqui, Waleska T Caiaffa, Nancy Lopez-Olmedo, Kevin Martinez-Folgar, J Jaime Miranda, Daniel A Rodriguez, Alejandra Vives, Ana V Diez-Roux

**Affiliations:** a Department of Epidemiology and Biostatistics, Urban Health Collaborative, Drexel Dornsife School of Public Health, Philadelphia, PA, USA; b Instituto de Salud Colectiva, Universidad Nacional de Lanús, Buenos Aires, Argentina; c Observatório de Saúde Urbana de Belo Horizonte, Universidade Federal de Minas Gerais, Belo Horizonte, Brazil; d Center for Population Health Research, National Institute of Public Health, Cuernavaca, Morelos, Mexico; e Instituto de Nutrición de Centroamérica y Panamá (INCAP), Guatemala; f CRONICAS Center of Excellence in Chronic Diseases and School of Medicine, Universidad Peruana Cayetano Heredia, Lima, Peru; g Department of City and Regional Planning and Institute for Transportation Studies, University of California Berkeley, Berkeley, CA, USA; h Departamento de Salud Pública, Escuela de Medicina, and CEDEUS, Pontificia Universidad Católica de Chile, Santiago, Chile

## Abstract

**Background:**

Latin America is one of the most unequal regions in the world, but evidence is lacking on the magnitude of health inequalities in urban areas of the region. Our objective was to examine inequalities in life expectancy in six large Latin American cities and its association with a measure of area-level socioeconomic status.

**Methods:**

In this ecological analysis, we used data from the Salud Urbana en America Latina (SALURBAL) study on six large cities in Latin America (Buenos Aires, Argentina; Belo Horizonte, Brazil; Santiago, Chile; San José, Costa Rica; Mexico City, Mexico; and Panama City, Panama), comprising 266 subcity units, for the period 2011–15 (expect for Panama city, which was for 2012–16). We calculated average life expectancy at birth by sex and subcity unit with life tables using age-specific mortality rates estimated from a Bayesian model, and calculated the difference between the ninth and first decile of life expectancy at birth (P90–P10 gap) across subcity units in cities. We also analysed the association between life expectancy at birth and socioeconomic status at the subcity-unit level, using education as a proxy for socioeconomic status, and whether any geographical patterns existed in cities between subcity units.

**Findings:**

We found large spatial differences in average life expectancy at birth in Latin American cities, with the largest P90–P10 gaps observed in Panama City (15·0 years for men and 14·7 years for women), Santiago (8·9 years for men and 17·7 years for women), and Mexico City (10·9 years for men and 9·4 years for women), and the narrowest in Buenos Aires (4·4 years for men and 5·8 years for women), Belo Horizonte (4·0 years for men and 6·5 years for women), and San José (3·9 years for men and 3·0 years for women). Higher area-level socioeconomic status was associated with higher life expectancy, especially in Santiago (change in life expectancy per P90–P10 change unit-level of educational attainment 8·0 years [95% CI 5·8–10·3] for men and 11·8 years [7·1–16·4] for women) and Panama City (7·3 years [2·6–12·1] for men and 9·0 years [2·4–15·5] for women). We saw an increase in life expectancy at birth from east to west in Panama City and from north to south in core Mexico City, and a core-periphery divide in Buenos Aires and Santiago. Whereas for San José the central part of the city had the lowest life expectancy and in Belo Horizonte the central part of the city had the highest life expectancy.

**Interpretation:**

Large spatial differences in life expectancy in Latin American cities and their association with social factors highlight the importance of area-based approaches and policies that address social inequalities in improving health in cities of the region.

**Funding:**

Wellcome Trust.

## Introduction

Latin America has large income inequalities, with eight countries located in the region being among the 20 countries with the highest income inequality worldwide.^1^ Moreover, social inequalities linked to other factors such as race or Indigenous origin,^2^ gender,^3^ and education^4^ are also prevalent. These social inequalities manifest themselves as large health inequalities, including differences in mortality and life expectancy at birth.

In addition to being highly unequal, the Latin American region is one of the most urbanised regions in the world, with over 500 million people, or 80% of the region's population, estimated to live in cities.^5^ Spatial segregation by socioeconomic position or race and ethnicity can lead to large spatial variations in health in cities. However, spatial inequalities in health in the large cities of Latin America have rarely been described or quantified.

Describing the magnitude of inequalities in life expectancy at birth in large Latin American cities is crucial to characterising and understanding the determinants of urban health in the region. In their latest strategic plan, published in October, 2019, the Pan American Health Organization (PAHO) placed health equity at the “heart of health” and stated that progress towards health equity is hindered by a “lack of consistent disaggregated data to track and reveal disparities”.^6^

Similar descriptive analyses in the cities of high-income countries have frequently been used to advocate for greater attention to health inequalities. For example, inequalities in life expectancy at birth across stations on the Jubilee line of the London Underground (London, UK)^7^ have been cited in many policy and media reports. During the most recent municipal election in Madrid, Spain, several candidates commented on inequalities in life expectancy at birth across neighbourhoods in the city, and the topic received extensive media coverage.^8^ Placing health inequalities at the centre of political discourse can support advocacy and the multisectoral policies needed to address them.

**Research in context**

**Evidence before this study**

Previous studies in the USA and Europe have shown inequalities in life expectancy by area in cities and that a lack of data on health inequalities, and subsequent lack of awareness of their existence, is a barrier to the design and implementation of policies to reduce them. Some of these studies have been used as powerful advocacy tools to raise awareness about the issue. Latin America is one of the most unequal regions in the world, yet evidence is lacking on the magnitude of these inequalities in urban areas of the region.

**Added value of this study**

To our knowledge, this study is the first to compare the life expectancy at birth in Latin American cities. A wide gap in life expectancy at birth exists in the six large Latin American cities we analysed: Buenos Aires, Argentina; Belo Horizonte, Brazil; Santiago, Chile; San José, Costa Rica; Mexico City, Mexico; and Panama City, Panama. We found cities with both wider and narrower gaps in life expectancy at birth than US metropolitan areas of a similar size. Spatial patterns in life expectancy varied by city, and we found an overall strong association with subcity unit-level socioeconomic status, proxied by educational attainment. Approaches to reduce health inequalities require data on their magnitude and distribution. This study provides some of the first estimates of the magnitude of inequalities in these six cities, inhabited by more than 50 million people overall.

**Implications of all the available evidence**

The presence of large special inequalities in health in large cities of Latin America highlights the fundamental role of social inequalities, residential segregation, and place-based factors in driving population health in the region. This evidence emphasises the potential crucial role of policies to reduce inequalities in urban areas, and might also be used as advocacy tools to bring social justice to people in cities in Latin America.

To our knowledge, no study has compared health inequalities in cities across a sample of several cities in Latin America. To address this gap, we used unique data compiled and harmonised by the Salud Urbana en America Latina (SALURBAL) study, a collaboration of 15 institutions in 11 countries in Latin America.^9^ We aimed to examine inequalities and spatial patterns of life expectancy at birth in six large Latin American cities and the extent to which this in-city inequality is associated with the socioeconomic status of the populations residing in these areas.

## Methods

### Study setting

In this ecological analysis, we used data from the SALURBAL study, which has compiled and harmonised data on health, social, and built environment for all cities with a population of more than 100 000 people in 11 countries: Argentina, Brazil, Chile, Colombia, Costa Rica, El Salvador, Guatemala, Mexico, Nicaragua, Panama, and Peru.^10^ Cities were defined as agglomerations of administrative units (ie, *municipios, comunas, partidos, delegaciones, cantones*, or *corregimientos*) that are covered, at least in part, by the urban extent of the city; more details are available elsewhere.^10^ For this study, we used data on six of the largest cities of the study: Buenos Aires, Argentina; Belo Horizonte, Brazil; Santiago, Chile; San José, Costa Rica; Mexico City, Mexico; and Panama City, Panama. We selected these cities for their large number of subcity units to allow in-city comparisons and the quality of vital registration systems. A definition of the subcity units for each city is listed in the appendix (p 2).

### Data sources

We used mortality and population data for the years 2011–15 in all cities, except for Panama City for which we used data for 2012–16. We obtained mortality data from vital registration systems in each country. Mortality records included data on at least sex and age at death, along with year of death and place of residence at time of death. Population data were obtained from national census bureaus (or equivalent) that provided population projections or estimations every year from 2011 to 2016 by age and sex for each subcity unit, as applicable to data availability per city.^10^ We obtained data on area-level socioeconomic status indicators from the latest available national census (ranging 2010–11 for all cities, apart from Santiago, Chile, for which data from the 2002 census were used because the 2012 census did not account for a substantial part of the population; appendix [p 2]) and we harmonised these data such that secondary education was equivalent across all countries.^10^ We obtained data on the proportion of each subcity unit that is built-up—ie, covered by an area classified as urbanised—from the Global Urban Footprint project.^10^

### Data handling

We used four variables from mortality records: age, sex, year of death, and location of residence at time of death. We categorised age in 5-year age groups (eg, 0–4, 5–9, 10–14), and capped the groups at age 75 years, since it was the upper limit at which age data in population projections were available for Costa Rica. We corrected data for the lack of complete registrations of all deaths (undercounting) using two death-distribution methods:^11^, ^12^ generalised growth balance and synthetic extinct generations,^11^ both implemented using the DDM R package. We calculated the harmonic mean of coverage estimates obtained from the two methods, an approach suggested to better account for existing migration flows to cities.^13^, ^14^ We truncated all coverage estimates at 1 (ie, any value above 1 was set to 1, and any value below 1 was left as supplied). We then applied a correction factor of (1/coverage) to the age-specific death count for each subcity unit. More details on how we estimated the coverage estimates along with a description of the coverage of death counts per subcity unit and city are in the appendix (pp 11–13).

To proxy socioeconomic status, we calculated the proportion of people aged 25 years or older who had completed secondary education or above in each subcity unit. To test whether associations were specific for education as a proxy in sensitivity analyses, we also calculated two more indicators of area-level socioeconomic status: proportion of households with water in the dwelling and the proportion of households that are overcrowded (ie, more than three people per room). For the three indicators, we used census data harmonised using the Integrated Public Use Microdata Series project (IPUMS) international recode.^15^

### Statistical analysis

For each city, we did analyses at the subcity-unit level. To avoid large fluctuations due to small units, we smoothed age-specific mortality rates using a Bayesian Poisson model, stratified by sex and city. This model included a random effect that was specific for each combination of subcity unit and age group, allowing for a first-order autoregressive correlation structure in the random effects that captured correlations between age-groups. These Bayesian estimates should, as a set, be less biased than estimates derived from observed data alone when some subcity units are small. We estimated age-specific mortality rates using the Markov Chain Monte Carlo method, with a burn-in period of 5000 iterations and 20 000 iterations after that. For each subcity unit, we sampled 2000 age and sex-specific mortality rates from the 20 000 iterations, and estimated 2000 values for life expectancy at birth by sex and subcity unit using life tables we constructed.^16^ To facilitate comparison with other studies, we extrapolated mortality rates to ages older than 85 years, using a Gompertz approximation^16^ with data from individuals aged 45–70 years.

For descriptive purposes, we calculated the median life expectancy at birth for each sex in each subcity unit and used it in all subsequent descriptions. To estimate the amount of variability in life expectancy at birth in and between cities, we ran a linear random intercept model of subcity units nested in cities and calculated the intraclass correlation coefficient, stratified by sex. To estimate the gap in life expectancy at birth, we calculated the difference in life expectancy at birth between the ninth and first deciles (P90–P10) of the subcity units in each city. We decided to use the P90–P10 gap because it is expressed in the original scale (years of life expectancy), presenting a measure of inequality that is interpretable and easy to communicate. We also estimated two other measures of inequality: the Gini coefficient and the coefficient of variation.^17^ We also mapped life expectancy at birth for each subcity unit for men and women using ArcGIS v10.4.

To estimate whether in-city inequality in life expectancy at birth is explained by differences in socioeconomic status at the subcity-unit level, we did a linear regression in which we modelled life expectancy at birth for each subcity unit as a function of subcity unit socioeconomic status, stratified by city. Interpretation of the coefficient is the difference in life expectancy at birth associated with a P90–P10 increase in the measure of socioeconomic status. To address heterogeneity in the urbanisation level of each subcity unit, we adjusted all models for the proportion of the subcity unit that is built-up. To acknowledge the uncertainty in the estimation of life expectancy at birth, we ran this model 2000 times (with the 2000 estimated life expectancies for each subcity unit), and then coefficients were pooled using Rubin's formula.^18^ All analyses, both descriptive and regression, were stratified by sex and weighted by average population in the subcity unit in the study period.

To test the robustness of these results to our analytical choices, we did five sensitivity analyses by repeating the entire set of analyses with five modifications. First, we only included subcity units with an estimated coverage of death counts equal to or higher than 90% and did not correct for undercounting in them. Second, we applied the correction factors, per subcity unit, without truncating them at 1. Third, we used life expectancy at age 40 years and 60 years to avoid potential uncorrected biases in the estimation of infant mortality (a strong determinant of life expectancy at birth) and to avoid issues with migration flows (more common in people aged 15–25 years). Fourth, we explored the association with two other proxies for area-level socioeconomic status: proportion of households with access to piped water in the dwelling, and proportion of households with overcrowding (ie, more than three people per room). Finally, we explored whether spatial autocorrelation might have affected our estimates of the association of socioeconomic status and life expectancy at birth by adjusting our analyses for the latitude and longitude (and their squared terms and their interaction) of the centroids of each subcity unit.^19^

We did all analyses in R version 3.5.1 and we implemented the Bayesian model in JAGS.

### Role of the funding source

The funder of the study had no role in study design, data collection, data analysis, data interpretation, or writing of the report. The corresponding author had full access to all the data in the study and had final responsibility for the decision to submit for publication.

## Results

All cities had at least 20 subcity units, ranging from 21 in Belo Horizonte to 76 in Mexico City (table 1). The median subcity population varied from 35 000 people (IQR 17 000–50 000) in Panama City to a high of 235 000 people (171 000–341 000) in Buenos Aires. The median subcity area varied from 24 km^2^ (5–53) in Panama City to a high of 196 km^2^ (72–304) in Belo Horizonte.

**Table 1:** Population, area, and educational attainment in six cities in Latin America and their corresponding subcity units

	**Buenos Aires, Argentina**	**Belo Horizonte, Brazil**	**Santiago, Chile**	**San José, Costa Rica**	**Mexico City, Mexico**	**Panama City, Panama**
**City**						
Total population, millions	15·3	5·0	6·4	2·5	20·6	1·9
Total area, km^2^	9982·1	4615·9	2427·8	3115·3	7819·1	3392·3
Total education*	36%	43%	47%	43%	40%	54%
**Subcity units**						
Number of units	51	21	36	29	76	53
Population, thousands	235 (171–341)	62 (29–170)	142 (101–211)	59 (42–85)	99 (29–404)	35 (17–50)
Area, km^2^	63 (20–229)	196 (72–304)	17 (11–60)	48 (25–168)	78 (39–145)	24 (5–53)
Education*	29% (24–44)	44% (35–52)	41% (36–52)	45% (37–51)	38% (32–45)	48% (40–64)

For subcity units data are median (IQR).

* Proportion aged ≥25 years who completed secondary education or above.

For men, average life expectancy at birth varied from a low of 69·9 years in Mexico City, to a high of 76·8 years in Panama City (table 2). However, wide variability was seen within cities, with P90–P10 gaps of 15·0 years in Panama City, 10·9 years in Mexico City, and 8·9 years in Santiago. By contrast, Buenos Aires, Belo Horizonte, and San José had narrower gaps of around 4 years. For women, Mexico City had the lowest average life expectancy at birth at 75·2 years, while Panama City had the highest at 86·1 years. Life expectancy at birth also varied widely within cities for women, with P90–P10 gaps of 17·7 years in Santiago, 14·7 years in Panama City, and 9·4 years in Mexico City. Again, the narrowest P90–P10 gaps were in Buenos Aires (5·8 years), Belo Horizonte (6·5 years), and San José (3·0 years). We found an intraclass correlation coefficient of 36·7% for men and 40·9% for women, meaning that most of the variability was within cities rather than between cities. We found that three measures of inequality (Gini coefficient, coefficient of variation, and the P90–P10 gap) were highly correlated with each other (Spearman's ρ >0·98; appendix p 4).

**Table 2:** Variability in life expectancy at birth and association with education in six large Latin American cities, by sex

	**Buenos Aires, Argentina**	**Belo Horizonte, Brazil**	**Santiago, Chile**	**San José, Costa Rica**	**Mexico City, Mexico**	**Panama City, Panama**
**Men**						
Life expectancy at birth, years	72·5	71·3	76·0	76·6	69·9	76·8
P90–P10 (gap), years	70·4 to 74·8 (4·4)	68·7 to 72·7 (4·0)	72·3 to 81·2 (8·9)	74·5 to 78·5 (3·9)	66·2 to 77·1 (10·9)	71·3 to 86·3 (15·0)
Change in life expectancy with education (95% CI), years*	3·5 (2·2 to 4·7)	4·4 (1·2 to 7·6)	8·0 (5·8 to 10·3)	0·6 (−1·3 to 2·6)	2·3 (0·3 to 4·2)	7·3 (2·6 to 12·1)
**Women**						
Life expectancy at birth, years	80·3	81·2	82·8	83·5	75·2	86·1
P90–P10 (gap), years	77·1 to 82·8 (5·8)	76·7 to 83·2 (6·5)	78·0 to 95·7 (17·7)	81·9 to 84·9 (3·0)	71·6 to 81·0 (9·4)	80·3 to 95·0 (14·7)
Change in life expectancy with education (95% CI), years*	3·7 (2·2 to 5·1)	5·3 (1·3 to 9·2)	11·8 (7·1 to 16·4)	0·7 (−1·6 to 3·0)	2·9 (1·1 to 4·7)	9·0 (2·4 to 15·5)

P90–P10=life expectancy at birth between the ninth and first deciles of subcity units.

* Change in years of life expectancy associated with a change in the proportion of people aged ≥25 years with completed secondary education or above equivalent to the P90–P10.

**Figure 1:**
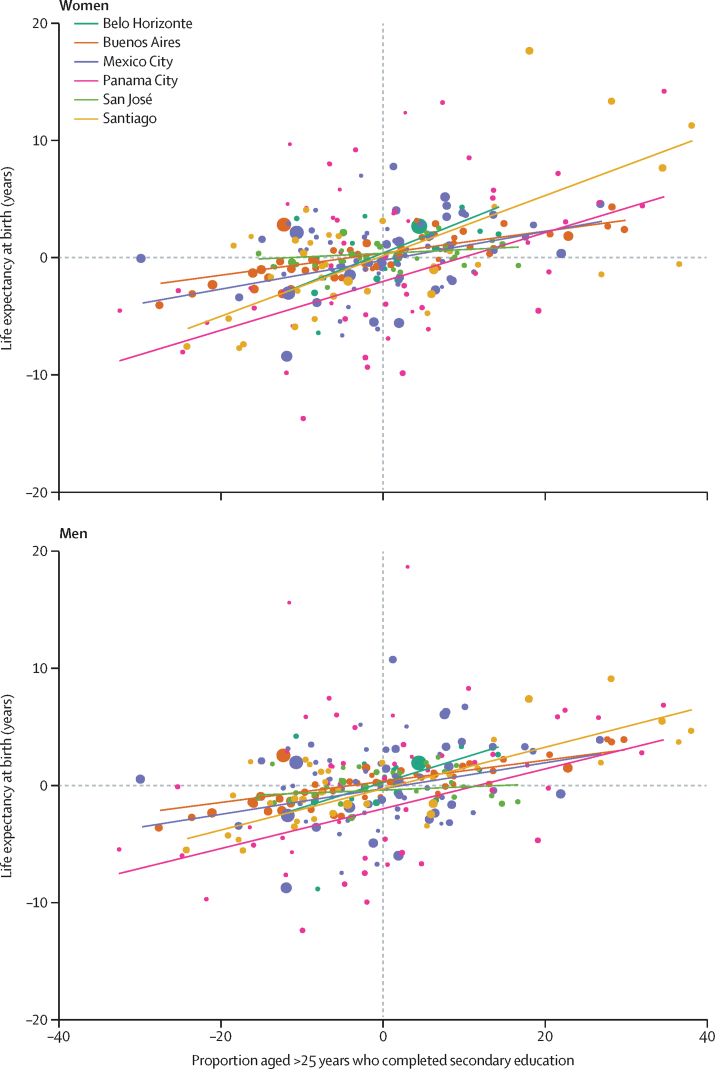
Association of life expectancy at birth with socioeconomic status, as proxied by educational attainment, in six large Latin American cities, adjusted for the proportion of subcity unit that is built-up, by sex Datapoint size is proportional to subcity unit population. Lines are linear regressions of life expectancy on education attainment, weighted by population and adjusted by proprotion of subcity unit that is built-up. The variables represented in the x axis and y axis are residuals of a regression, at the city level, of educational attainment (x axis) or life expectancy (y axis) on the proportion of the subcity unit that is built-up.

For Santiago and Panama City, a change in subcity unit-level educational attainment equivalent to the P90–P10 gap was associated with an increase in life expectancy at birth of 7–12 years for men and women (table 2, figure 1). This association was lower in magnitude in Belo Horizonte, Buenos Aires, and Mexico City. The association was the weakest in San José, with a less than 1-year increase per one-unit increase in education; this association was similar for both men and women (figure 1).

**Figure 2:**
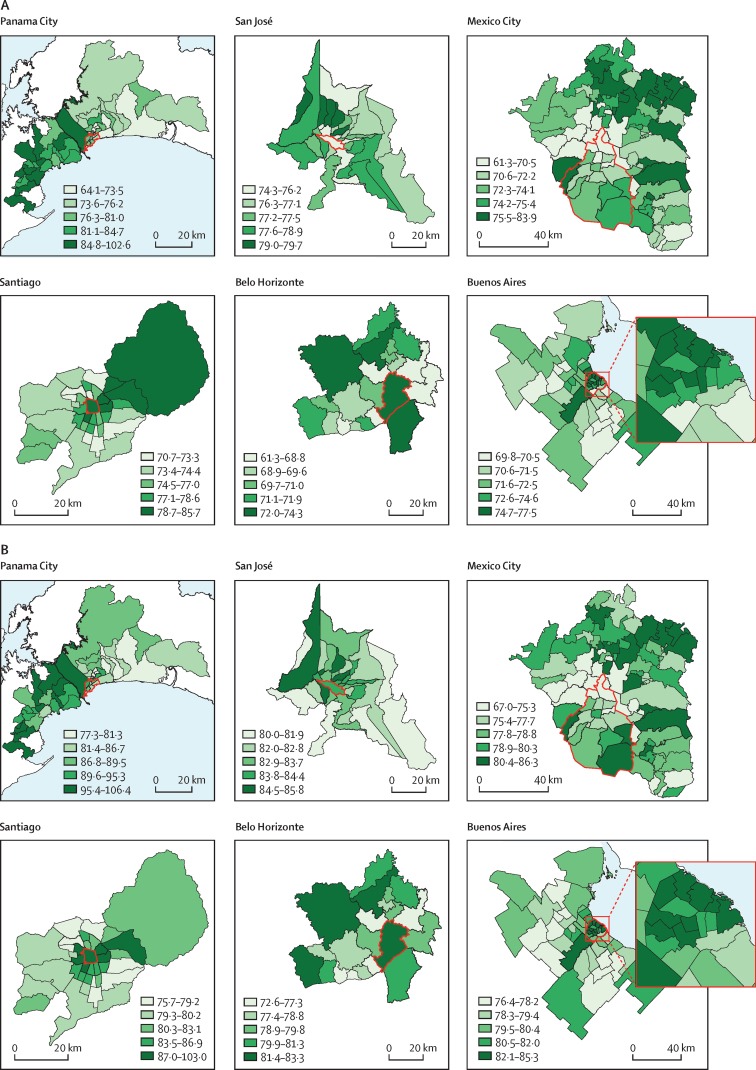
Spatial distribution of life expectancy at birth in men (A) and women (B) in six Latin American cities Maps of cities with subcity units indicated. Categories are quintiles of life expectancy at birth in each city. Red lines outline the 11 central *corregimientos* of Panama City, the central *distrito* of San José, the 16 *delegaciones* of Mexico City, the central *comuna* of Santiago, the central *municipio* of Belo Horizonte, and the 15 *comunas* of the Ciudad Autonoma de Buenos Aires (also shown in the inset).

The variability in life expectancy at birth is not random and we observed important geographical patterns in cities between subcity units (figure 2). In Panama City, a higher life expectancy at birth was seen in the western part of the city than in the other areas of the city. A similar pattern was seen in Mexico City, where the northern part of the core city and the adjacent areas in the metropolitan area have a lower life expectancy at birth than other areas. Santiago and Buenos Aires have a mixed pattern, with both a core-periphery divide (higher life expectancy in the core part of both cities than elsewhere) and an increasing west-to-east pattern seen in Santiago and an increasing south-to-north pattern seen in the core of Buenos Aires. The central *comuna* of Santiago has the highest life expectancy at birth in the city, followed by the *comunas* to the east. For Buenos Aires, the northern *comunas* of the Ciudad Autonoma de Buenos Aires (and adjacent *partidos* of the Provincia de Buenos Aires) have a higher life expectancy at birth compared with both the southern-central *comunas* and the southern-peripheral areas. In San José, the central part of the city has low life expectancy at birth, and the periphery is divided into areas of high and low life expectancy at birth with no clear pattern. Belo Horizonte has a mixed pattern, with the core subcity unit (the *municipio* of Belo Horizonte) having the highest life expectancy at birth.

Sensitivity analyses using life expectancy at age 40 years and at age 60 years as the outcomes, water access and overcrowding as the exposures, changing the specifications of the undercounting correction methods, and adjusting for spatial autocorrelation showed similar inferences to our main results (appendix pp 5–10). An interactive app is available online with versions of figure 1 with the variations of all sensitivity analyses along with detailed data on the variables used in this study.

## Discussion

Our study has shown large variability in life expectancy at birth in six large Latin American cities—Buenos Aires, Belo Horizonte, Santiago, San José, Mexico City, and Panama City—large spatial inequalities in life expectancy at birth, and an association with area-level socioeconomic status. The spatial variability in life expectancy at birth differed substantially between cities, as did the extent to which subcity unit-level socioeconomic status was associated with life expectancy at birth. Inequalities were largest in Panama City, Santiago, and Mexico City, while the association with subcity unit-level socioeconomic status was strongest in Santiago and Panama City. We also found distinct spatial patterns of life expectancy at birth in every city. While the differences between the city with the highest (Panama City) and lowest (Mexico City) average life expectancy at birth were approximately 7 years for men and 11 years for women, this difference was overshadowed in both cities by a P90–P10 gap of 9·4–15·0 years in life expectancy at birth in the subcity units across both sexes.

A few reports from high-income countries have described variations in life expectancy at birth or survival in cities or across small areas;^7^, ^20^, ^21^, ^22^ however, to our knowledge, no other study has described variations in life expectancy at birth within multiple Latin American cities. A previous study in the core areas of Buenos Aires found a similar gradient in all-cause and cause-specific mortality as we found here.^23^ Other studies in single cities in the USA and Europe have also found similar gaps. For instance, using data from the Global Burden of Disease study, the P90–P10 gap in census tracts in King County, WA, USA, was calculated as 8·3 years in men and 6·2 years in women,^22^ 11 years for both sexes combined in communities in Chicago, IL, USA,^21^ and 11 years for both sexes combined in community statistical areas of Baltimore, MD, USA.^24^ A study in Rotterdam, Amsterdam, found that the total inequality in life expectancy at birth between neighbourhoods was around 6 years for both men and women,^20^ while a study of inequalities in life expectancy at birth in London, UK, found a 20-year range in areas of around of 7000 people.^7^ These results in London have been used as advocacy tools,^7^ while much narrower gaps in Madrid, Spain, were used as part of the political discussion leading up to the 2015 local elections.^8^ However, our results regarding the size of inequality are difficult to compare with previous work because the geographical units we used were large and heterogeneous, which will likely result in narrower gaps. However, to our knowledge, no other study has compared gaps in life expectancy between multiple cities in any region, including Latin America. Our study serves as a benchmark for other studies looking at these gaps in other regions or contexts.

We found heterogeneity in spatial and socioeconomic inequality in life expectancy at birth, and at least four factors potentially contribute to this heterogeneity. First, socioeconomic status might be a strong predictor of life expectancy at birth but spatial segregation varies across cities. Cities with less economic spatial segregation will have a weaker association of area-level socioeconomic status with life expectancy at birth than those with more economic segregation. As such, one would also expect to see smaller spatial inequalities in life expectancy at birth. Second, variations in the measurement of the indicator for socioeconomic status—educational attainment. Although we applied the IPUMS international recode,^15^ different educational systems might lead to heterogeneity in the indicator. However, in our sensitivity analysis, looking at other subcity unit-level proxies for socioeconomic status (water access and overcrowding) we found analogous results. Third, variations in the measurement of the outcome. Lack of complete coverage of deaths is an endemic issue in many Latin American countries.^12^, ^25^ However, we applied state-of-the-art methods to account for this phenomenon and selected cities with more than 90% coverage. Nevertheless, the possibility remains that our correction did not entirely solve this issue, but our sensitivity analyses using variations of these methods rendered similar inferences. Finally, area-level socioeconomic status might have a differential association with mortality by country. For instance, previous research has shown narrower education gradients in Costa Rica and Mexico than in the USA.^26^, ^27^ Additional work is needed to confirm these large differences in the degree of spatial patterning and in the associations of area-level life expectancy at birth with area-level socioeconomic status, and to investigate the reasons for these differences if they are confirmed.

Our study has several strengths. First, we have compiled and harmonised data across six different cities, ensuring the comparability of both exposures and outcomes. Second, we corrected for the lack of complete coverage using state-of-the-art demography methods at the subcity-unit level. Third, we selected cities that had a relatively high number of subcity units to enable observation of gaps in life expectancy at birth. Finally, to avoid issues with small areas we used a Bayesian Poisson model that derived improved estimates of rates for areas with small populations.

Our study also has several limitations. First, the definition of a subcity unit varies by country. Since the definition of a subcity unit leads to differences in who gets included in each unit, this definition will modify the width of the gap in life expectancy at birth and probably the association between education and life expectancy at birth. Second, some of the units are small, which leads to increased fluctuations in life expectancy at birth. We addressed this limitation by generating Bayesian estimates that smooth mortality rates towards the city-level mean and towards closer age groups. This method might have reduced the variability in life expectancy at birth in cities, therefore our estimates are a conservative measure of the health inequalities in these six cities. Third, our correction for undercounting might not be complete. However, given that lack of coverage might be associated with socioeconomic status (because coverage is increased in high socioeconomic status areas)^14^ our results would represent a conservative estimate of the inequality in life expectancy at birth by education because life expectancy at birth might be overestimated in lower socioeconomic areas. Finally, our estimates of socioeconomic status rely on the latest available census before the years of the mortality data. For Santiago, Chile, the census data we used were from the census in 2002 because the 2012 census did not account for a substantial part of the population.^28^ However, in a post-hoc analysis, we tested whether any changes were seen in the educational attainment of subcity units in Santiago by harmonising educational attainment indicators^10^ for the 2002 and 2017 census, and comparing the proportion of people with secondary education or above in all subcity units of Santiago; we found a very high correlation between 2002 and 2017 (ρ=0·97).

Approaches to reduce health inequalities require data on their magnitude and distribution. Awareness about specific areas with higher health needs might improve resource reallocation or other sorts of place-based public policies. Previous research has shown that a lack of data on health inequalities, and subsequent lack of awareness of their existence, is a barrier on the design and implementation of policies to reduce them.^29^ Future research should expand this effort to more cities with a large number of subcity units, or collect and analyse data at smaller units of analysis (eg, census tracts) where available, allowing for a better and finer characterisation of the segregation patterns in life expectancy at birth across Latin American cities. SALURBAL^9^, ^10^ will be exploring variations between smaller areas (which are likely to be much larger than those reported here) when detailed geocoded mortality data become available.

Our study showed a wide gap in life expectancy in six large Latin American cities, different segregation patterns (north to south, east to west, or core periphery) in each city, and an association with subcity unit-level socioeconomic status. These results might also be used as advocacy tools for political incidence in bringing social justice to cities in Latin America.
