# Correction to *Lancet Planet Health* 2019; 3: e503–10

**DOI:** 10.1016/S2542-5196(19)30265-7

**Published:** 2020-01-03

*Bilal U, Alazraqui M, Caiaffa WT, et al. Inequalities in life expectancy in six large Latin American cities from the SALURBAL study: an ecological analysis.* Lancet Planet Health *2019;* 3: *e503–10*—In this Article, due to a linkage error with the Panama City data, life expectancy data, P90–P10 gaps, and associations with education have been corrected for Panama City in the Summary, table 2, the Results, where appropriate in the Discussion, in figures 1 and 2, and in the appendix. Additionally, support from the SALURBAL investigators has been added to the Acknowledgments section. These corrections have been made as of Jan 3, 2020.

