# Supplementary material for: Inequalities in life expectancy in six large Latin American cities from the SALURBAL study: an ecological analysis
Source: Lancet Planet Health. 2019 Dec;3(12):e503–10. doi: 10.1016/S2542-5196(19)30235-9 (PMC6926471; doi:10.1016/S2542-5196(19)30235-9)
Supplement: Supplementary appendix [file mmc1.pdf]

THE LANCET
Planetary Health

Supplementary appendix

This appendix formed part of the original submission and has been peer reviewed. We post it as supplied by the authors.

Supplement to: Bilal U, Alazraqui M, Caiaffa WT, et al. Inequalities in life expectancy in six large Latin American cities from the SALURBAL study: an ecological analysis. *Lancet Planet Health* 2019; published online Dec 10. http://dx.doi.org/10.1016/S2542-5196(19)30235-9.

Table of Contents:

Supplementary Appendix, Part 1:

- Supplementary Table 1: Definition of sub-city units and timeline of data availability
- Supplementary Figure 1: Detailed distribution of life expectancy and socioeconomic status indicators within cities.
- Supplementary Figure 2: Correlations between different measures of inequality
- Supplementary Table 3: Results with Life Expectancy at Age 40
- Supplementary Table 4: Results with Life Expectancy at Age 60
- Supplementary Table 5: Results with Water Access and Overcrowding
- Supplementary Table 6: Results including only sub-city units with coverage >=90%.
- Supplementary Table 7: Results without truncation of correction factors at 1.
- Supplementary Table 8: Results adjusting for latitude, longitude, latitude2, longitude2, and latitude x longitude, of the centroid of each sub-city unit.

Supplementary Appendix, Part 2:

- Methods to account for the lack of complete coverage of mortality / undercounting of deaths

Note: supplementary data and the results of the sensitivity analyses in figure form are available at: https://usamabilal.shinyapps.io/MS33_LE

Supplementary

Supplementary Table 1: Definition of sub-city units and timeline of data availability

City	# Units	Definition of sub-city units	Mortality and Population Data	Census Data (Education)
Buenos Aires, AR	51	15 *Comunas* (Ciudad Autónoma de Buenos Aires) and 36 *Partidos* (Provincia de Buenos Aires)	2011-2015	2010
Belo Horizonte, BR	21	21 *Municipios*	2011-2015	2010
Santiago, CL	36	36 *Comunas*	2011-2015	2002
San Jose, CR	29	29 *Cantones*	2011-2015	2011
Mexico City, MX	76	16 *Delegaciones* (Ciudad de México) and 60 *Municipios* (surrounding áreas, part of the Estado de Mexico)	2011-2015	2010
Panama City, PA	53	53 *Corregimientos*	2012-2016	2010

Footnote: in all cases, the entire city is defined by an agglomeration of the sub-city units that are part of the built-up area of the city (as defined by satellite imagery).

Supplementary Figure 1: Detailed distribution of population, area, life expectancy, coverage, and socioeconomic status indicators between and within the cities.

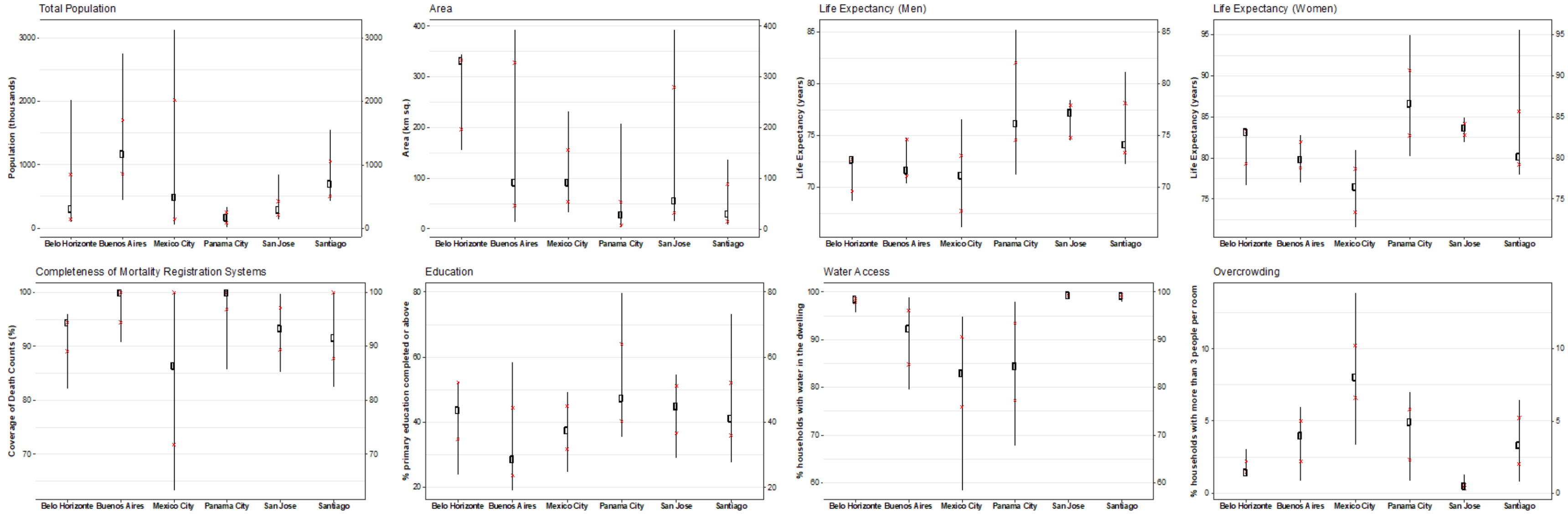

Footnote: The lines represent the 10th and 90th percentile, the red crosses the 25th and 75th percentile, and the dot is the 50th percentile (median). All (except total population) are weighted by the population of the sub-city.

Detailed data by sub-city unit is available at: https://usamabilal.shinyapps.io/MS33_LE
Please note that due to confidentiality concerns given the size of some sub-city units, we have only identified the city they belong to, and have not included identifiers on sub-city units themselves. We have also rounded population numbers to the closest thousand to avoid identification.

Supplementary Figure 2: correlations between 3 measures of inequality (Gini coefficient, Coefficient of Variation, P90P10 gap)

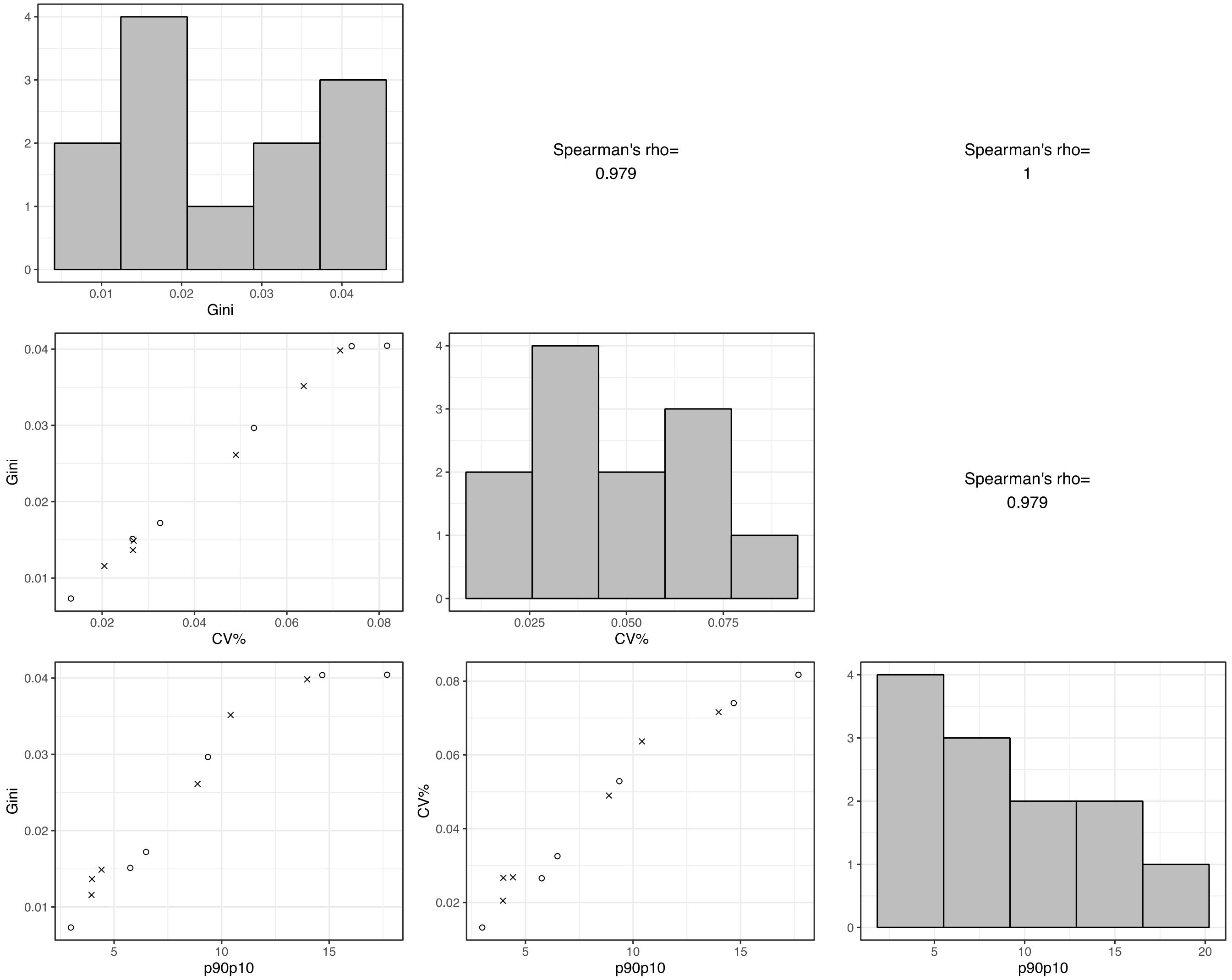

Footnote: each unit is a city, with crosses for men and circles for women.

Supplementary Table 3: Results with Life Expectancy at Age 40

	Men			Women		
**City**	**City LE40**	**P90-P10 (difference)**	**Education Slope**	**City LE40**	**P90-P10 (difference)**	**Education Slope**
Buenos Aires, AR	35.2	33.3-37.0 (3.6)	2.6 (1.6;3.6)	42.1	39.1-44.3 (5.2)	3.2 (1.8;4.5)
Belo Horizonte, BR	36.1	33.7-37.1 (3.3)	2.7 (0.0;5.4)	43.4	39.2-45.3 (6.2)	4.8 (0.9;8.8)
Santiago, CL	38.0	35.0-42.8 (7.8)	7.3 (5.0; 9.6)	44.1	39.3-57.3 (17.9)	11.6 (6.9;16.3)
San Jose, CR	39.2	37.4-41.0 (3.6)	0.1 (-1.7;1.9)	45.2	43.5-46.5 (3.0)	0.5 (-1.8;2.8)
Mexico City, MX	34.3	31.9-40.7 (8.8)	1.4 (-0.2;2.9)	37.8	35.0-43.0 (8.0)	2.6 (1.0;4.1)
Panama City, PA	40.0	35.3-48.9 (13.6)	5.4 (1.0; 9.7)	47.8	42.3-56.2 (13.9)	8.2 (1.7;14.7)

Supplementary Table 4: Results with Life Expectancy at Age 60

City	Men			Women		
	**City LE60**	**P90-P10 (difference)**	**Education Slope**	**City LE60**	**P90-P10 (difference)**	**Education Slope**
Buenos Aires, AR	18.1	16.5-19.5 (3.1)	1.9 (1.0;2.7)	24.2	21.4-26.1 (4.6)	2.7 (1.4;4.0)
Belo Horizonte, BR	20.1	18.0-20.9 (2.9)	1.7 (-1.0;4.5)	25.9	21.9-27.7 (5.8)	4.4 (0.1;8.7)
Santiago, CL	20.4	17.1-24.8 (7.7)	6.0 (3.5;8.4)	25.7	20.8-38.5 (17.8)	11.0 (6.1;15.8)
San Jose, CR	21.9	20.4-23.6 (3.2)	-0.4 (-2.3;1.5)	26.8	24.9-28.3 (3.3)	0.2 (-2.2;2.7)
Mexico City, MX	18.0	15.7-23.4 (7.7)	0.9 (-0.4;2.3)	20.3	17.7-25.2 (7.5)	2.1 (0.7;3.6)
Panama City, PA	22.5	18.9-30.8 (11.9)	4.5 (0.2;8.8)	29.4	23.6-37.5 (13.9)	7.5 (0.9;14.1)

Supplementary Table 5: Results with Water Access and Overcrowding

	Men		Women	
City	Water Slope	Overcrowding Slope	Water Slope	Overcrowding Slope
Buenos Aires, AR	2.2 (0.7;3.8)	-3.1 (-4.6;-1.5)	3.1 (1.3;4.8)	-3.7 (-5.4;-2.0)
Belo Horizonte, BR	1.3 (-0.2;2.8)	-2.7 (-5.0;-0.3)	0.8 (-1.2;2.8)	-2.3 (-5.3;0.7)
Santiago, CL	1.4 (-1.2;3.9)	-6.0 (-8.8;-3.1)	2.4 (-2.1;7.0)	-9.1 (-14.4;-3.7)
San Jose, CR	0.7 (-0.9;2.2)	-1.7 (-4.1;0.7)	0.6 (-1.2;2.5)	0.0 (-2.9;3.0)
Mexico City, MX	2.3 (-0.2;4.9)	-1.6 (-4.0;0.7)	2.4 (0.0;4.9)	-2.3 (-4.5;-0.1)
Panama City, PA	5.0 (-0.7;10.6)	-4.3 (-9.2;0.5)	7.4 (-0.2;15.1)	-6.1 (-12.6;0.4)

Note: water refers to % households with access to piped water in the dwelling. Overcrowding refers to % of households with more than 3 people per room.

Supplementary Table 6: Results including only sub-city units with coverage >=90%.

			Men			Women		
City	# Units	Ed. P90-P10	City LEB	P90-P10 (difference)	Education Slope	City LEB	P90-P10 (difference)	Education Slope
Buenos Aires, AR	49	39.2%	72.9	71.1-75.8 (4.7)	3.8 (2.5;5.1)	80.8	77.9-83.8 (5.9)	4.2 (2.7;5.7)
Belo Horizonte, BR	10	17.4%	73.1	70.3-73.7 (3.4)	1.2 (-2.4;4.9)	83.5	78.0-84.4 (6.4)	0.9 (-4.7;6.4)
Santiago, CL	22	45.2%	78.2	74.4-84.1 (9.7)	6.8 (3.9; 9.7)	86.8	80.3-98.9 (18.6)	10.9 (4.7;17.0)
San Jose, CR	20	25.7%	78.0	75.3-80.2 (4.9)	0.8 (-2.2;3.9)	84.4	82.5-86.0 (3.5)	1.4 (-2.2;5.0)
Mexico City, MX	57	38.9%	74.2	70.6-80.1 (9.5)	3.1 (0.2;5.9)	79.4	77.1-83.9 (6.8)	4.2 (2.0;6.3)
Panama City, PA	42	46.2%	77.5	73.3-84.8 (11.5)	3.0 (-3.1;9.1)	86.9	82.4-96.0 (13.6)	4.5 (-4.1;13.2)

Supplementary Table 7: Results without truncation of correction factors at 1.

		Men			Women		
**City**	**Ed. P90-P10**	**City LEB**	**P90-P10 (difference)**	**Education Slope**	**City LEB**	**P90-P10 (difference)**	**Education Slope**
Buenos Aires, AR	39.4%	73.0	70.4-74.9 (4.5)	3.6 (2.4;4.8)	80.9	77.4-83.2 (5.8)	3.9 (2.4;5.4)
Belo Horizonte, BR	28.1%	71.3	68.7-72.7 (4.0)	4.4 (1.1;7.7)	81.2	76.7-83.2 (6.5)	5.3 (1.3;9.3)
Santiago, CL	45.4%	76.5	72.3-83.7 (11.4)	11.2 (7.7;14.7)	83.6	78.0-100.6 (22.6)	17.1 (10.1;24.1)
San Jose, CR	25.8%	76.7	74.5-78.4 (3.9)	0.6 (-1.3;2.6)	83.6	82.0-84.9 (2.8)	0.7 (-1.6;3.0)
Mexico City, MX	24.4%	70.4	66.2-84.6 (18.5)	1.9 (-0.8;4.7)	75.6	71.6-88.0 (16.4)	2.6 (-0.1;5.2)
Panama City, PA	44.1%	77.9	71.3-88.1 (16.9)	7.7 (2.4;13.0)	87.6	80.3-100.6 (20.2)	9.9 (2.2;17.6)

Supplementary Table 8: Results adjusting for latitude, longitude, latitude^2, longitude^2, and latitude x longitude, of the centroid of each sub-city unit.

City	Education Slope (Men)	Education Slope (Women)
Buenos Aires, AR	4.8 (3.5;6.1)	4.8 (3.2;6.3)
Belo Horizonte, BR	4.5 (1.6;7.4)	4.3 (0.8;7.8)
Santiago, CL	8.6 (5.7;11.6)	13.5 (7.1;20.0)
San Jose, CR	0.6 (-1.7;3.0)	-0.1 (-2.9;2.7)
Mexico City, MX	3.9 (1.7;6.2)	4.1 (2.1;6.1)
Panama City, PA	7.0 (3.2;10.9)	8.0 (2.1;13.8)

Methods to account for the lack of complete coverage of mortality / undercounting of deaths

1. Introduction

Vital registration systems, especially those of lower quality, do not cover all deaths that occur in their territory. This leads to a phenomenon of undercounting, where not all deaths are counted, and therefore leads to an underestimation of mortality rates (and overestimation of life expectancy). In the Latin American region, this issue is especially salient in Peru, Colombia and Central American countries. To diagnose and correct this issue, there are demographic methods that can estimate the degree of undercounting (or coverage), and allow for the upward correction of death counts.

2. Methods

We have implemented Death Distribution Methods[1-4]. These methods compare the age distribution between two time points (generally two censuses), and then check whether the number of deaths registered matches what would be expected. There are two methods that have been found as the most robust: the generalized growth balance method (GGB) and the synthetic extinct generations method (SEG), and a hybrid method that combines both (GGB-SEG). More details on these methods can be found elsewhere[1-4]. We implemented these methods using the DDM R package[5].

These methods have similar assumptions, namely: (1) population is closed to migration; (2) undercounting is constant by age; and (3) no error in age reporting. While (2) and (3) may be of concern, their effect may be minimal compared to (1) [closure to migration] in the context of cities. Given strong rural to urban, and urban to urban migration flows, the estimation of death coverage in cities is challenging. To account for this, we have implemented several strategies.

3. Strategies to account for migration

Among the strategies used to account for migration are: (1) obtaining data on migration and correcting the methods above accordingly[6]; (2) calculating coverage in an age band that is less affected by migration[2]; and (3) leveraging different methods that respond differentially to migration (for example, one is biased downwards and the other is biased upwards)[2,4]. Given the challenging nature of obtaining migration data for all cities in our sample, we have opted to implement (2) and (3).

Following Hill[2], we have calculated the mean (harmonic, appropriate for ratios) of the generalized growth balance (GGB) and synthetic extinct generations (SEG) methods. Following Peralta et al[4] we have selected the best-fitting age-band as calculated by the R DDM package. This finds the age band in which the root mean squared error is minimized. With this, we obtain an estimation of coverage that we use to correct death counts. We limit these estimates at 1 or below, so that death counts can only be corrected upward.

We have implemented two sensitivity analyses:

1. Not correcting coverage but only including sub-city areas with estimated >=90% coverage
2. Not truncating coverage estimates at 1, so that death counts may also be corrected downwards.

4. Data sources

There are two key data sources needed to implement these methods:

- Population counts: we obtained population counts at the sub-city level from population projections[7] in 2011 and 2015 (2012-2016 for Panama City), by 5-year age group.
- Death counts: we obtained death counts at the sub-city level from vital registration systems[7] in 2011 through 2015 (2012-2016 for Panama City), by 5-year age group.

5. Results

We estimated the following coverage of death counts for each sub-city unit. The black cross (X) shows the estimated coverage for each city as a whole.

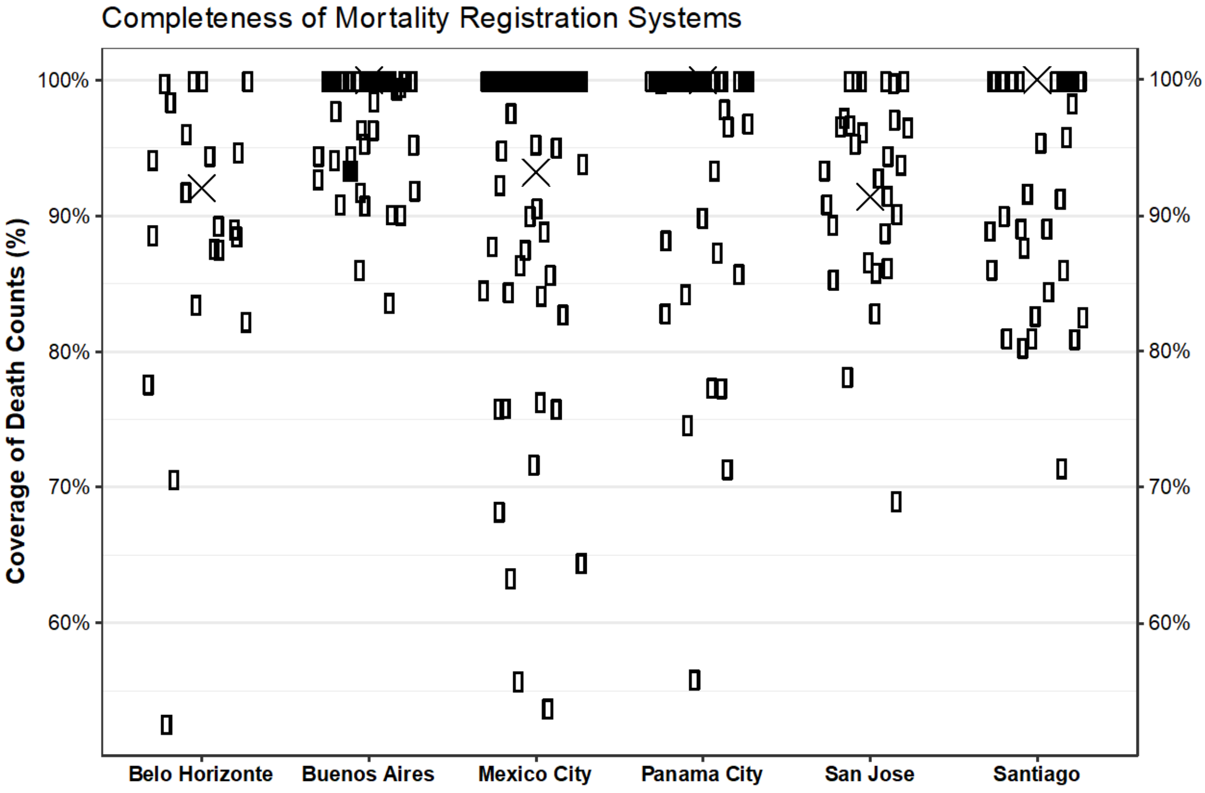

References

1. Adair T, Lopez AD. Estimating the completeness of death registration: An empirical method. PloS one 2018;13:e0197047.

2. Hill K, You D, Choi Y. Death distribution methods for estimating adult mortality: sensitivity analysis with simulated data errors. Demographic Research 2009;21:235-54.

3. Hill K. Analytical methods to evaluate the completeness and quality of death registration: Current state of knowledge. Population Division Technical Paper 2017.

4. Peralta A, Benach J, Borrell C, et al. Evaluation of the mortality registry in Ecuador (2001–2013) – social and geographical inequalities in completeness and quality. Population health metrics 2019;17:3.

5. Riffe T, Lima EECd, Queiroz B. DDM: Death Registration Coverage Estimation (R Package). 2017.

6. Hill K, Queiroz B. Adjusting the general growth balance method for migration. Revista Brasileira de Estudos de População 2010;27:7-20.

7. Quistberg DA, Diez Roux AV, Bilal U, et al. Building a Data Platform for Cross-Country Urban Health Studies: the SALURBAL Study. Journal of urban health : bulletin of the New York Academy of Medicine 2018.
